# Pragmatic trials may help to identify effective strategies to reduce nursing home antipsychotic medication use

**DOI:** 10.1186/s13584-016-0130-3

**Published:** 2017-01-26

**Authors:** Rosa R. Baier, Vincent Mor

**Affiliations:** Brown University School of Public Health, Box G-S121-6, 121 S. Main Street, Providence, RI 02912 USA

**Keywords:** Antipsychotic medications, Dementia, Nursing homes, Chemical restraints, Quality

## Abstract

Despite widespread agreement that nursing homes’ use of antipsychotic medications for residents without specific psychiatric diagnoses is a marker of poor quality of care, prevalence remains high. Additionally, variation suggests continued opportunity to improve care even in countries, like the United States, that have long-standing policies designed to decrease antipsychotic medication use. In a recent Israel Journal of Health Policy Research article, Frankenthal et al. presented results linking increased antipsychotic medication use prevalence in Tel Aviv nursing homes with facility characteristics, including some that “undermine quality of care,” and called for increased national focus on this area. While we agree with the authors that government focus can help to decrease antipsychotic medication use, experience in the United States shows that such efforts may not be sufficient: we present data showing significant variation among United States nursing homes’ antipsychotic medication use prevalence after more than ten years of national warnings and programs. This suggests that United States nursing home clinicians and caregivers continue to need effective non-pharmacologic interventions to substitute for antipsychotic medications. We suggest expanded use of cluster-randomized trials to test strategies to withdraw residents from antipsychotic medications and to implement alternate, non-pharmacological approaches for addressing the behavioral and psychological symptoms of dementia.

## Main text

The off-label use of antipsychotic medications to address the behavioral and psychological symptoms of dementia is controversial, because antipsychotic medications may increase older adults’ risk of heart failure, sudden death, and pneumonia [1, 2]. As a result, nursing homes’ use of antipsychotics as chemical restraints for the purpose of behavioral control is widely considered inappropriate, absent a psychiatric diagnosis, and is increasingly considered to be a marker of poor quality of care. Despite general agreement that non-pharmacological approaches are preferable, studies in Europe [3], Canada [4], the United States [5, 6], and elsewhere show that antipsychotic medication use remains highly prevalent in this setting with evidence of substantial variation between facilities [5, 7] and between countries [3, 8].

Understanding this variation can inform programs and policies intended to reduce antipsychotic medication use. The SHELTER study compared treatment of nursing home residents in seven European countries and Israel, and found that 32% of residents with a dementia diagnosis had an antipsychotic prescription; prevalence ranged from 18% in Israel to 60% in the Czech Republic [3]. However, this study only included a relatively small number of volunteer facilities in each country, with considerable intra-country variation. In a recent Israel Journal of Health Policy Research (IJHPR) article, Frankenthal et al. [7] presented results linking increased APM prevalence among Tel Aviv nursing homes with facility characteristics, including some that “undermine quality of care.” These included the presence of medical directors without specialized geriatrics training, shortages of social workers and occupational therapists, and the use of unsafe or non-fitting self-aid equipment [7]. The authors argue that their findings shed light on a problematic care process, making it transparent in a country where antipsychotic medication use is not regulated or publicly reported, and call for increased national focus on this area.

**Fig. 1:**
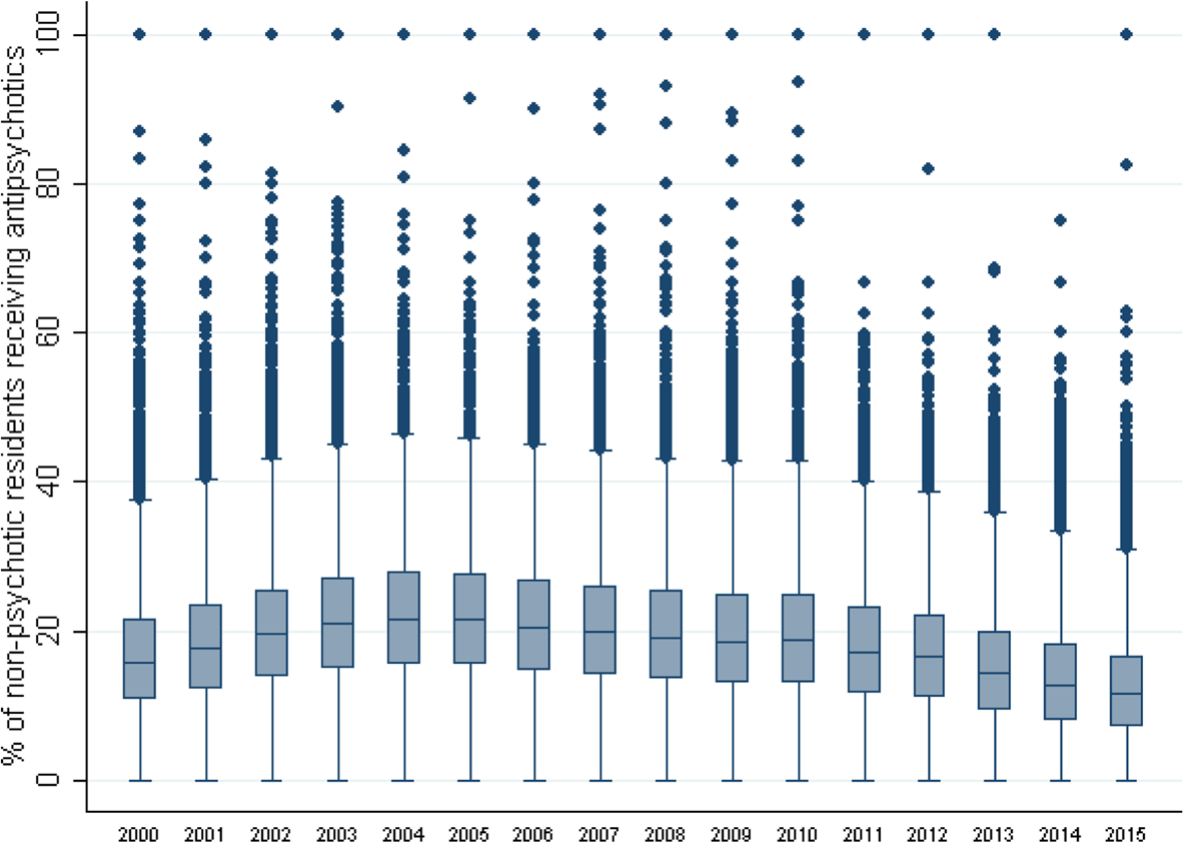
Percent of United States nursing home residents on antipsychotic medications without a psychiatric diagnosis, 2000–2010

In the United States, there has been a national spotlight on reducing nursing home antipsychotic medication use since our country’s Food and Drug Administration issued a “boxed warning” against the use of these medications for off-label indications among older adults, in 2005 [1]. More recently, the Centers for Medicare & Medicaid Services began publicly reporting nursing home antipsychotic medication rates on its Nursing Home Compare website (in 2011) [9] and implemented a national campaign to lower use (in 2012) [10]. Although national prevalence has decreased over the past 10 years, our analysis of longitudinal data available through Brown University’s LTCFocus.org database [11] illustrates significant, ongoing variation in facility-level antipsychotic medication prevalence (Fig. 1) and suggests continued need for intervention in the United States. While there are many nursing facilities with no residents (or virtually none) taking antipsychotics, even in 2014 there were hundreds of facilities with 40% or more of their residents prescribed these medications.

To reduce nursing home antipsychotic medication use, clinicians must implement strategies to identify and discontinue inappropriate off-label use among residents with existing prescriptions [12] and practices to avoid new prescriptions when the behavioral and psychological symptoms of dementia become manifest [13]. Both approaches require alternative techniques so that clinicians and caregivers have strategies to implement in lieu of antipsychotic medications if dementia behaviors re-emerge or persist. Although there are numerous studies evaluating various non-pharmacological strategies to reduce antipsychotic medications use or address dementia behaviors, much of the evidence for the effectiveness of these interventions is insufficient [14]. Furthermore, complex, labor-intensive interventions that might substitute for antipsychotics can be difficult to implement with existing staff and resources. Indeed, many interventions that reveal a positive effect in small- scale studies implemented by research staff have difficulty showing similar effects in real-world replications. Additionally, some researchers argue that rigorously testing such interventions may be difficult, in part because it is not possible to blind nursing home clinicians to interventions.

Cluster-randomized trial methods are one methodological approach to overcome research limitations for testing strategies to reduce antipsychotic medication use or improve the behavioral and psychological symptoms of dementia using non-pharmacological approaches. By randomizing NHs to implement antipsychotic medication practice guidelines, for example, or to serve as control sites, we can rigorously test such interventions using pragmatic methods. At Brown University, we use cluster-randomized trials to conduct studies on topics such as influenza vaccination [15, 16]; others have used these methods for antipsychotic medication reduction interventions [17–19]. The Brown studies pair cluster-randomized trial methods with the use of existing clinical and administrative data, such as United States Minimum Data Set resident assessment data; this allows us to conduct rigorous nursing home research efficiently. We suggest that others use similar approaches and data sources, e.g., the Minimum Data Set or InterRAI assessment tools [20], to test antipsychotic medication interventions using approaches that mimic real-world conditions. One such trial is currently underway in Ontario, offering a model for others around the developed world [21].

## Conclusions

Despite widespread agreement that nursing homes’ use of antipsychotic medications as chemical restraints is associated with poor clinical outcomes and as such constitutes a marker of poor quality of care, prevalence remains high and variation suggests continued opportunity to improve care even in countries, like the United States, that have long-standing policies designed to decrease APM use. While we agree with Frankenthal et al. [7] that regulation and public reporting can help to decrease prevalence, the United States experience suggests that nursing home clinicians and caregivers also need effective alternative interventions to employ as substitutes for antipsychotics. We suggest expanded use of cluster-randomized trials to test strategies to remove residents from antipsychotics and to implement alternate, non-pharmacological approaches for addressing the behavioral and psychological symptoms of dementia.

## References

[1] U.S. Department of Health & Human Services, U.S. Food and Drug Administration. Public health advisory: deaths with antipsychotics in elderly patients with behavioral disturbances. 2005. Available from: http://www.fda.gov/Drugs/DrugSafety/PostmarketDrugSafetyInformationforPatientsandProviders/ucm053171.htm. Accessed 25 July 2016.

[2] Lapeyre-Mestre M (2016). A review of adverse outcomes associated with psychoactive drug use in nursing home residents with dementia. Drugs Aging.

[3] Foebel AD, Liperoti R, Onder G, Finne-Soveri H, Henrard JC, Lukas A, Denkinger MD, Gambassi G, Bernabei R (2014). SHELTER study investigators. Use of antipsychotic drugs among residents with dementia in european long-term care facilities: results from the SHELTER study. J Am Med Dir Assoc.

[4] Foebel A, Ballokova A, Wellens NI, Fialova D, Milisen K, Liperoti R, Hirdes JP (2015). A retrospective, longitudinal study of factors associated with new antipsychotic medication use among recently admitted long-term care residents. BMC Geriatr.

[5] Chen Y, Briesacher BA, Field TS, Tjia J, Lau DT, Gurwitz JH (2010). Unexplained variation across US nursing homes in antipsychotic prescribing rates. Arch Intern Med.

[6] Maust DT, Langa KM, Blow FC, Kales HC. Psychotropic use and associated neuropsychiatric symptoms among patients with dementia in the USA. Int J Geriatr Psychiatry. 2016. [Epub ahead of print]. 10.1002/gps.4452 PMC4990518 26889640

[7] Frankenthal D, Zandman-Goddard G, Ben-Muvhar Y, Porat-Katz BS (2016). The impact of facility characteristics on the use of antipsychotic medications in nursing homes: a cross-sectional study. Isr J Health Policy Res.

[8] Feng Z, Hirdes JP, Smith TF, Finne-Soveri H, Chi I, Du Pasquier JN, Gilgen R, Ikegami N, Mor V (2009). Use of physical restraints and antipsychotic medications in nursing homes: a cross-national study. Int J Geriatr Psychiatry.

[9] Centers for Medicare & Medicaid Services (CMS). Nursing home compare datasets. Available from: https://data.medicare.gov/data/nursing-home-compare. Accessed 25 July 2016.

[10] Centers for Medicare & Medicaid Services (CMS). National partnership to improve dementia care in nursing homes. 2015. Available from: https://www.cms.gov/Medicare/Provider-Enrollment-and-Certification/SurveyCertificationGenInfo/National-Partnership-to-Improve-Dementia-Care-in-Nursing-Homes.html. Accessed 25 July 2016.

[11] Brown school of public health. shaping long-term care in america project. Available from: http://ltcfocus.org/. Accessed 25 July 2016.

[12] Azermai M (2015). Dealing with behavioral and psychological symptoms of dementia: a general overview. Psychol Res Behav Manag.

[13] Tjia J, Reidenberg MM, Hunnicutt JN, Paice K, Donovan JL, Kanaan A, Briesacher BA, Lapane KL (2015). Approaches to gradual dose reduction of chronic Off-label antipsychotics used for behavioral and psychological symptoms of dementia. Consult Pharm.

[14] Jutkowitz E, Brasure M, Fuchs E, Shippee T, Kane RA, Fink HA, Butler M, Sylvanus T, Kane RL (2016). Care-delivery interventions to manage agitation and aggression in dementia nursing home and assisted living residents: a systematic review and meta-analysis. J Am Geriatr Soc.

[15] Gravenstein S, Dahal R, Gozalo PL, Davidson HE, Han LF, Taljaard M, Mor V (2016). A cluster randomized controlled trial comparing relative effectiveness of two licensed influenza vaccines in US nursing homes: Design and rationale. Clin Trials.

[16] Gravenstein S, Taljaard M, Gozalo P, Dahal R, Davidson HE, Han L, Ogarek J, Mor V. Relative effect of high-dose influenza vaccination on hospitalizations of older adults in United States nursing homes: results from a cluster-randomized controlled trial. Open Forum Infect Dis. 2015;2(Suppl 1): LB-8.

[17] Tjia J, Field T, Mazor K, Lemay CA, Kanaan AO, Donovan JL, Briesacher BA, Peterson D, Pandolfi M, Spenard A, Gurwitz JH (2015). Dissemination of evidence-based antipsychotic prescribing guidelines to nursing homes: a cluster randomized trial. J Am Geriatr Soc.

[18] Ballard C, Orrell M, YongZhong S, Moniz-Cook E, Stafford J, Whittaker R, Woods B, Corbett A, Garrod L, Khan Z, Woodward-Carlton B, Wenborn J, Fossey J (2016). Impact of antipsychotic review and nonpharmacological intervention on antipsychotic use, neuropsychiatric symptoms, and mortality in people with dementia living in nursing homes: a factorial cluster-randomized controlled trial by the well-being and health for people with dementia (WHELD) program. Am J Psychiatry.

[19] Pieper MJ, Francke AL, van der Steen JT, Scherder EJ, Twisk JW, Kovach CR, Achterberg WP (2016). Effects of a stepwise multidisciplinary intervention for challenging behavior in advanced dementia: a cluster randomized controlled trial. J Am Geriatr Soc.

[20] interRAI. Long-term care facilities (LTCF). 2016. Available from: http://interrai.org/long-term-care-facilities.html. Accessed 25 July 2016.

[21] Desveaux L, Gomes T, Tadrous M, Jeffs L, Taljaard M, Rogers J, Bell CM, Ivers NM (2016). Appropriate prescribing in nursing homes demonstration project (APDP) study protocol: pragmatic, cluster-randomized trial and mixed methods process evaluation of an Ontario policy-maker initiative to improve appropriate prescribing of antipsychotics. Implement Sci.

